# A Mendelian randomization study on the causal effects of cigarette smoking on liver fibrosis and cirrhosis

**DOI:** 10.3389/fmed.2024.1390049

**Published:** 2024-05-22

**Authors:** Liwei Guo, Yong An, Xu Huang, Wenhua Liu, Fangfang Chen, Yuchen Fan, Shuai Gao, Liyan Han, Kai Wang

**Affiliations:** ^1^Medical Integration and Practice Center, Shandong University, Jinan, Shandong, China; ^2^Department of Epidemiology, The First Affiliated Hospital of Shandong First Medical University and Shandong Provincial Qianfoshan Hospital, Jinan, Shandong, China; ^3^Department of Respiratory, The First Affiliated Hospital of Shandong First Medical University and Shandong Provincial Qianfoshan Hospital, Shandong Institute of Respiratory Diseases, Jinan, Shandong, China; ^4^Department of Hepatology, Qilu Hospital, Shandong University, Jinan, Shandong, China; ^5^Institute of Hepatology, Shandong University, Jinan, Shandong, China

**Keywords:** cigarette smoking, liver fibrosis, cirrhosis, Mendelian randomization, causality

## Abstract

**Background:**

Liver fibrosis significantly impacts public health globally. Untreated liver fibrosis eventually results in cirrhosis. Cigarette smoking is the main etiologic factor for various diseases. However, the causal effects of cigarette smoking on liver fibrosis and cirrhosis have yet to be fully elucidated.

**Methods:**

In this study, Mendelian randomization (MR) analysis was performed to assess the association between cigarette smoking, liver fibrosis, and cirrhosis. Single-nucleotide polymorphisms (SNPs) were selected as instrumental variables from a genome-wide association study (GWAS) of European ancestry. Patients were divided into six exposure categories as follows: “ever smoked,” “pack years of smoking,” “age of smoking initiation,” “smoking status: never,” “smoking status: current,” and “smoking status: previous.” The outcomes of this study included liver fibrosis and cirrhosis. MR-Egger, weighted median, inverse variance weighted, simple mode, and weighted mode were selected as the analysis methods. Cochran’s Q and the MR-PRESSO tests were conducted to measure heterogeneity. The MR-Egger method was performed to evaluate horizontal pleiotropy, while the “leave-one-out” analysis was performed for sensitivity testing.

**Results:**

The results of this study showed that having a smoking history increases the risk of liver fibrosis and cirrhosis [“ever smoked”: odds ratio (OR) = 5.704, 95% CI: 1.166–27.910, *p* = 0.032; “smoking status: previous”: OR = 99.783, 95% CI: 2.969–3.353e+03, *p* = 0.010]. A negative correlation was observed between patients who never smoked and liver fibrosis and cirrhosis (“smoking status: never”: OR = 0.171, 95% CI: 0.041–0.719, *p* = 0.016). However, there were no significant associations between “smoking status: current,” “pack years of smoking,” and “age of smoking initiation” and liver fibrosis and cirrhosis. Cigarette smoking did not have a significant horizontal pleiotropic effect on liver fibrosis and cirrhosis. The “Leave-one-out” sensitivity analysis indicated that the results were stable.

**Conclusion:**

The study confirmed the causal effects of cigarette smoking on liver fibrosis and cirrhosis.

## Introduction

Liver disease is a long-standing challenge to global health (1). The etiological factors for chronic liver inflammation include viral hepatitis infections, alcohol consumption, drugs, metabolic factors, and autoimmune hepatitis. Untreated chronic liver inflammation causes liver fibrosis. Advanced liver fibrosis results in cirrhosis and hepatocellular carcinoma (HCC). Liver cirrhosis is the 11th most frequent cause of death worldwide, with two million deaths every year, due to complications such as chronic portal hypertension, bleeding events, and hepatic encephalopathy (2, 3).

Cigarette smoking is associated with an unhealthy lifestyle. The number of smokers is rapidly increasing, and more than one billion people worldwide have a pernicious smoking habit (4). Cigarette smoke contains more than 4,000 toxic substances that are etiological factors for various diseases (5–8). Cigarette smoking is primarily associated with respiratory diseases, and it is a major risk factor for cardiovascular diseases (9–12). Additionally, cigarette smoking increases the risk of gastrointestinal disorders (13). Cigarette smoking is associated with an increased incidence of tumors, including liver cancer (14). In addition, the incidence of liver cancer in current smokers is 1.5 times higher than that in non-smokers (15, 16). However, the effect of cigarette smoking on liver fibrosis and cirrhosis remains unclear.

Mendelian randomization (MR) is a novel tool that uses summary statistics from genome-wide association studies (GWAS) to investigate the causality between risk factors and diseases (17, 18). In MR studies, genetic variations are used as instrumental variables (IVs) (19, 20). Randomized controlled trials (RCTs) are widely accepted as the gold standard for assessing the association between exposures and outcomes. However, RCTs have many limitations, including high costs and difficulties in implementation (21, 22). MR is an analog method for RCTs (23). Additionally, MR can overcome the deficiencies of RCTs through the use of single-nucleotide polymorphisms (SNPs) as IVs (18, 24, 25). MR studies can assess the causal effects of various exposures of interest, including biological markers, daily behaviors, and disease exposures, on a range of diseases (26–28).

Previous studies estimated the causal effects of cigarette smoking on many diseases, such as stroke and cancer, using MR (7, 29–31). However, studies using MR to assess the effect of cigarette smoking on liver fibrosis and cirrhosis are limited. Hence, this MR study aimed to clarify the causal effect of cigarette smoking on liver fibrosis and cirrhosis.

## Materials and methods

### Data sources

A two-sample MR analysis was used to explore the association between exposure and outcome in two different samples. Compared to a one-sample MR, the sample size in a two-sample MR is larger and more precise (32). This study aimed to analyze the causal effects of cigarette smoking on liver fibrosis and cirrhosis. As such, six exposures related to cigarette smoking were selected, including “ever smoked,” “pack years of smoking,” “age of smoking initiation,” “non-smokers,” “current smokers,” and “former smokers.” The “ever smoked” group included participants who ever had smoking habits, regardless of whether they were currently smoking or not. Pack years of smoking were calculated based on the age of starting smoking and the age of quitting smoking, or the duration from starting smoking to participating in this program of the Integrative Epidemiology Unit GWAS database. The “non-smokers” group included participants who had never smoked. The “current smokers” group consists of participants who had the smoking habit and currently still smoke. The “former smokers” group consists of participants who used to smoke before but had completely quit smoking recently. Compared to non-smokers, anyone who has a smoking habit before or currently is considered to have a smoking history.

The exposed genetic variants were obtained from the Integrative Epidemiology Unit GWAS database. The sample sizes for “ever smoked,” “pack years of smoking,” “age of smoking initiation,” “smoking status: never,” “smoking status: current,” and “smoking status: previous” were 461,066, 142,387, 341,427, 359,706, 336,024, and 336,024, respectively. The study focused on outcomes such as liver fibrosis and cirrhosis, and the genetic variants obtained from the FinnGen consortium data (1,602 cases and 332,951 controls) were also included in this study. To avoid population stratification, the genetic variants used in this analysis were derived from European ancestries. The details of the data sources are listed in Table 1. In this MR study, genetic variants strongly correlated with exposure but failed to show associations with confounders. Therefore, genetic variants did not have an impact on the outcome, except through exposure (33).

**Table 1:** Detailed information of datasets included for MR analysis.

Exposure	Consortium			Sample size	Population	Sex	Number of SNPs
Ever smoked	MRC-IEU			461,066	European	Males and Females	9,851,867
Pack years of smoking	MRC-IEU			142,387	European	Males and Females	9,851,867
Age of smoking initiation	GWAS and Sequencing Consortium of Alcohol and Nicotine use			341,427	European	Males and Females	11,894,779
Smoking status: never	Neale lab			359,706	European	Males and Females	13,586,591
Smoking status: current	Neale Lab			336,024	European	Males and Females	10,894,596
Smoking status: previous	Neale Lab			336,024	European	Males and Females	10,894,596
**Outcomes**	**Consortium**	**Cases**	**Control**	**Sample size**	**Population**	**Sex**	**Number of SNPs**
Liver fibrosis and cirrhosis	FinnGen	1,602	332,951	334,553	European	Males and Females	20,169,350

### SNP selection

SNPs with a *p*-value less than 5 × 10^−8^ and minor frequency > 1% as IVs, relating to “ever smoked,” “pack years,” “age of smoking initiation,” “non-smokers,” “current smokers,” and “former smokers,” were selected. Furthermore, the clumping method (r^2^ < 0.001, clumping distance = 10,000 kb) was used to avoid linkage disequilibrium. The F-statistic was used to evaluate the strength of the association between IVs and exposure. The general threshold of F in an MR study was 10 (31). SNPs with an F less than 10 were considered weak instruments and were eliminated from further MR analyses (32). In this study, F was calculated as β^2^/SE^2^ (β stands for the effect on the risk of exposure, and SE stands for the standard error) (34–36).

### Statistical analysis

MR-Egger, weighted median, inverse variance weighted (IVW), simple mode, and weighted mode were selected as the methods of analysis. IVW is considered the most reliable method in MR analyses, and it was performed to assess the heterogeneity among the IVs (37). Cochran’s Q and the MR-PRESSO tests were used to measure the heterogeneity of individual SNPs (38). Heterogeneity existed if the *p*-value was less than 0.05 and the random-effects model was implemented; otherwise, the fixed-effects model was used. Funnel plots were used to show heterogeneity by drawing Wald ratios for the SNPs. The MR-Egger method was used to evaluate horizontal pleiotropy; if the intercept was significantly different from 0, with a *p*-value less than 0.05, horizontal pleiotropic effects existed (39). Finally, sensitivity analyses were conducted using the “leave-one-out” analysis.

In this study, the statistically significant level was set at *p* < 0.05. All analyses were performed in R (version 4.2.2) with the “TwoSampleMR” package (version 0.5.6).

## Results

The SNPs chosen for this MR analysis are presented in Supplementary File. In the final MR analysis, a total of 67 SNPs were related to “ever smoked,” 5 SNPs were related to “pack years of smoking,” 9 SNPs were related to “age of smoking initiation,” 61 SNPs were related to “smoking status: never,” 15 SNPs were related to “smoking status: current,” and 18 SNPs were related to “smoking status: previous.” The F statistics for these SNPs were more than 10, with mean *F* values of 40.63, 37.62, 81.23, 41.77, 40.79, and 35.30 for “ever smoked,” “pack years of smoking,” “age of smoking initiation,” “smoking status: never,” “smoking status: current,” and “smoking status: previous,” respectively. Furthermore, a weak instrumental variable bias was non-existent.

**Figure 1:**
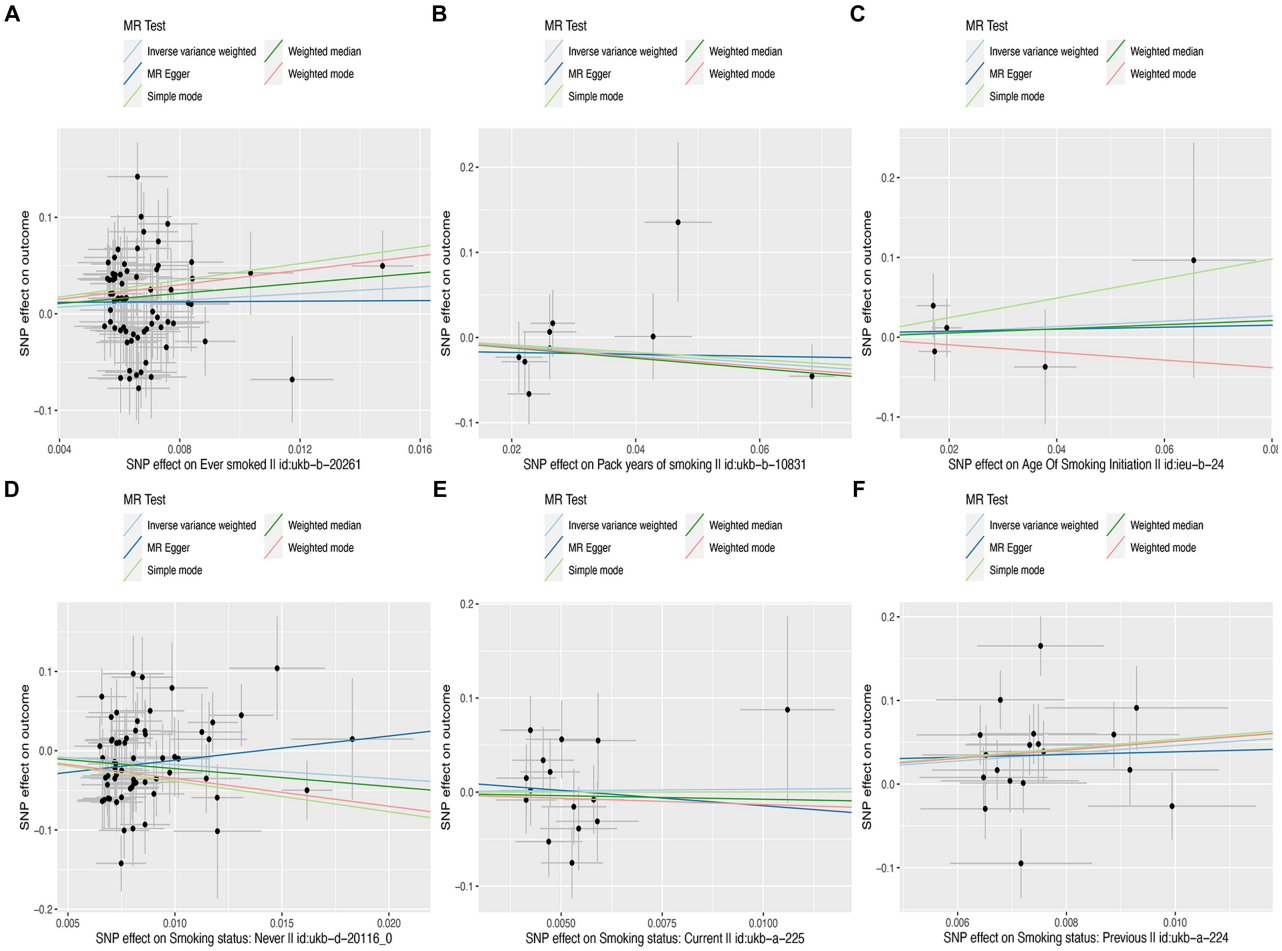
Scatter plots of effects of cigarette smoking-associated SNPs on liver fibrosis and cirrhosis. **(A)** “Ever smoked”; **(B)** “Pack years of smoking”; **(C)** “Age of smoking initiation”; **(D)** “Smoking status: never”; **(E)** “Smoking status: current”; and **(F)** “Smoking status: previous.”

**Figure 2:**
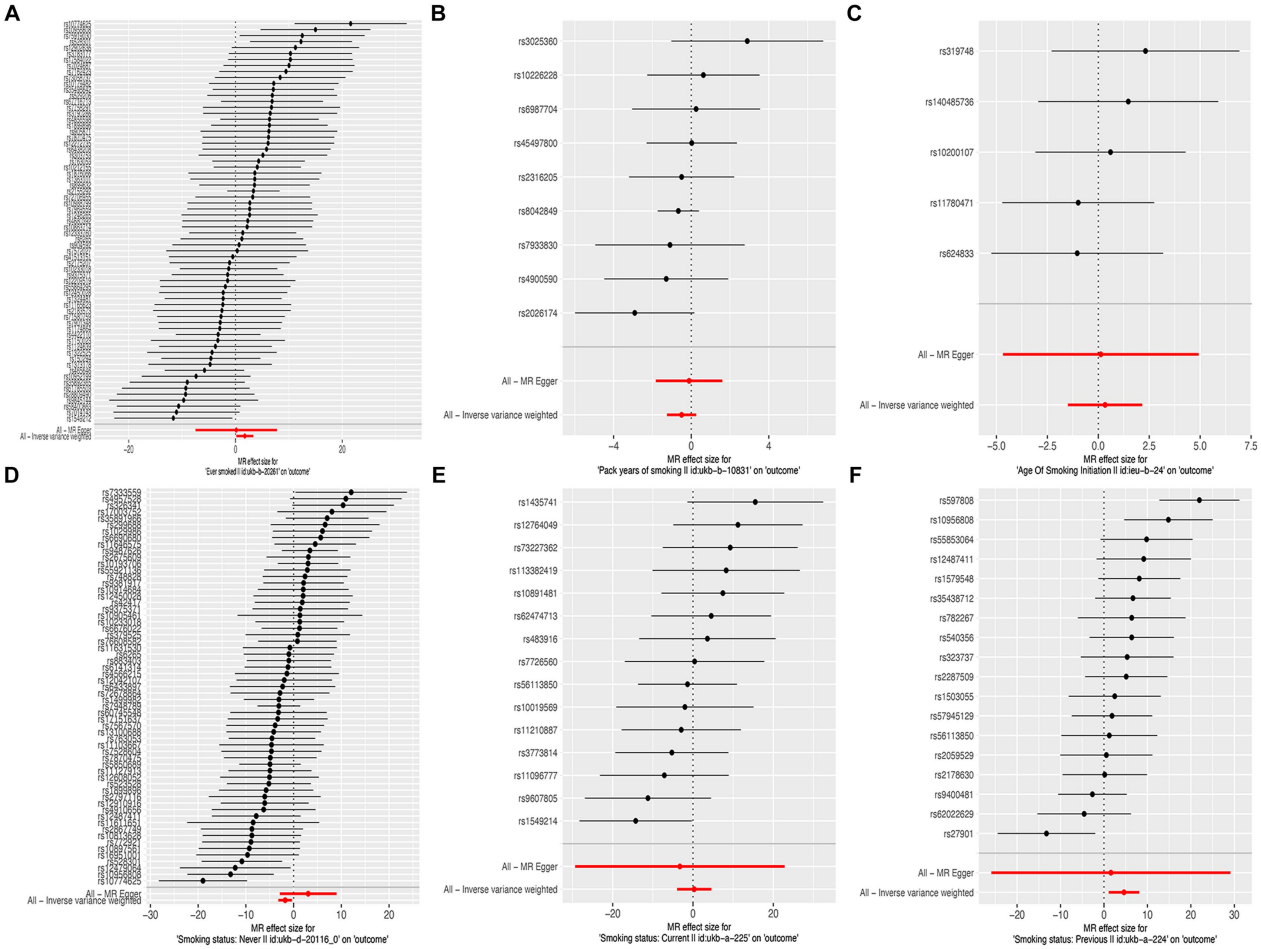
Forest plots of the effect size for each SNP for cigarette smoking on the risk of liver fibrosis and cirrhosis. **(A)** “Ever smoked”; **(B)** “Pack years of smoking”; **(C)** “Age of smoking initiation”; **(D)** “Smoking status: never”; **(E)** “Smoking status: current”; and **(F)** “Smoking status: previous.”

In this MR study, five methods were used to assess the causal effects of cigarette smoking on liver fibrosis and cirrhosis in the European population, and the IVW method was considered the most reliable method. As presented in Table 2, having a smoking history was correlated with liver fibrosis and cirrhosis (“ever smoked,” IVW: OR = 5.704, 95% CI: 1.166–27.910, *p* = 0.032). “Smoking status: previous” also had positive associations with liver fibrosis and cirrhosis (IVW: OR = 99.783, 95% CI: 2.969 – 3.353e + 03, *p* = 0.010). “Smoking status: never” had negative associations with liver fibrosis and cirrhosis (IVW: OR = 0.171, 95% CI: 0.04–0.719, *p* = 0.016). Additionally, “smoking status: current,” “pack years of smoking,” and “age of smoking initiation” were not associated with liver fibrosis and cirrhosis. The effect of each SNP on liver fibrosis and cirrhosis is shown in Figures 1, 2.

**Table 2:** MR analysis from each method assessing the causal effects of smoking on liver fibrosis and cirrhosis.

Outcome	Exposure	Method	OR(95%CI)	*p*-value
Fibrosis and cirrhosis of the liver	Ever smoked	MR Egger	1.160(0.001–2406.793)	0.970
		Weighted median	14.097(1.754–113.327)	0.013
		Inverse variance weighted	5.704(1.166–27.910)	0.032
		Simple mode	76.732(0.261–22556.380)	0.139
		Weighted mode	43.153(0.406–4587.957)	0.118
Fibrosis and cirrhosis of the liver	Pack years of smoking	MR Egger	0.895(0.161–4.970)	0.902
		Weighted median	0.544(0.217–1.359)	0.202
		Inverse variance weighted	0.608(0.286–1.294)	0.197
		Simple mode	0.647(0.143–2.935)	0.580
		Weighted mode	0.567(0.222–1.447)	0.297
Fibrosis and cirrhosis of the liver	Age of Smoking Initiation	MR Egger	1.134(0.009–138.311)	0.962
		Weighted median	1.298(0.136–12.370)	0.820
		Inverse variance weighted	1.396(0.225–8.665)	0.720
		Simple mode	3.407(0.159–72.768)	0.477
		Weighted mode	0.621(0.031–12.399)	0.770
Fibrosis and cirrhosis of the liver	Smoking status: never	MR Egger	21.316(0.056–8130.904)	0.317
		Weighted median	0.104(0.016–0.663)	0.017
		Inverse variance weighted	0.171 (0.041–0.719)	0.016
		Simple mode	0.021(0.000–1.898)	0.098
		Weighted mode	0.030(0.001–1.297)	0.073
Fibrosis and cirrhosis of the liver	Smoking status: current	MR Egger	0.038(1.752e-13-8.243e+09)	0.810
		Weighted median	0.469(1.654e-03-1.328e+02)	0.792
		Inverse variance weighted	1.366(1.879e-02-9.934e+01)	0.887
		Simple mode	1.014(2.569e-05-4.006e+04)	0.998
		Weighted mode	0.269(1.304e-05-5.545e+03)	0.799
Fibrosis and cirrhosis of the liver	Smoking status: previous	MR Egger	4.889(5.494e-12-4.351e+12)	0.911
		Weighted median	168.202(5.980–4.730e+03)	0.003
		Inverse variance weighted	99.783(2.969–3.353e+03)	0.010
		Simple mode	210.954(0.491–9.057e+04)	0.102
		Weighted mode	171.430(0.380–7.731e+04)	0.117

**Figure 3:**
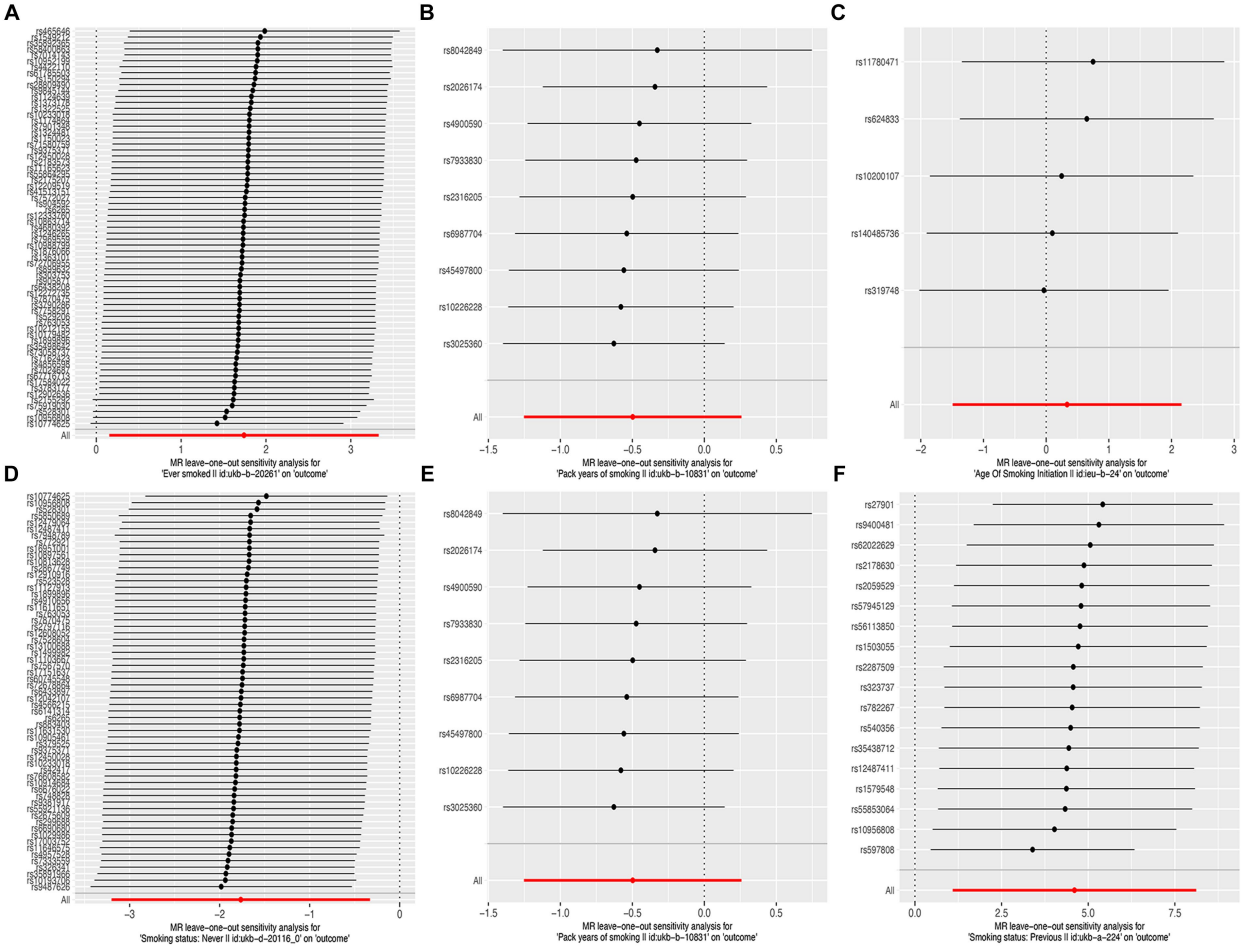
Leave-one-out sensitivity analysis of the causal effects of cigarette smoking on liver fibrosis and cirrhosis. **(A)** “Ever smoked”; **(B)** “Pack years of smoking”; **(C)** “Age of smoking initiation”; **(D)** “Smoking status: never”; **(E)** “Smoking status: current”; and **(F)** “Smoking status: previous.”

The results of the MR-Egger test indicated the absence of pleiotropy (p ˃0.05). In addition, all SNPs in this MR analysis did not affect liver fibrosis and cirrhosis via biological pathways independently (Table 3). Heterogeneity was tested using Cochran’s Q test and the MR-PRESSO test; the results are presented in Table 3. Funnel plots were used to visualize the heterogeneity of the effects of SNPs on liver fibrosis and cirrhosis; the results are presented in Supplementary Figure 1. The results of the leave-one-out analysis revealed reliable associations between exposures and outcomes (Figure 3).

**Table 3:** Pleiotropy and heterogeneity testing of smoking associated with liver fibrosis and cirrhosis risk using the MR Egger method.

Exposure	Method	Intercept	Standard error		*p*-value
Ever smoked	MR Egger	0.011	0.027		0.677
Pack years of smoking	MR Egger	−0.015	0.0315		0.638
Age of Smoking initiation	MR Egger	0.005	0.055		0.933
Smoking status: never	MR Egger	−0.043	0.026		0.107
Smoking status: current	MR Egger	0.018	0.067		0.789
Smoking status: previous	MR Egger	0.023	0.105		0.831
**Exposure**	**Method**	**Cochran’s Q test**			**MR-PRESSO test**
		**Q**	**df**	***p*-value**	**Global Test *p*-value**
Ever smoked	Inverse variance weighted	95.466	66	0.010	0.011
	MR Egger	95.210	65	0.008	
Pack years of smoking	Inverse variance weighted	6.634	8	0.577	0.661
	MR Egger	6.392	7	0.495	
Age of Smoking Initiation	Inverse variance weighted	1.870	4	0.760	0.779
	MR Egger	1.861	3	0.602	
Smoking status: never	Inverse variance weighted	89.178	60	0.009	0.0094
	MR Egger	85.298	59	0.014	
Smoking status: current	Inverse variance weighted	15.941	14	0.317	0.327
	MR Egger	15.850	13	0.257	
Smoking status: previous	Inverse variance weighted	37.988	17	0.002	0.004
	MR Egger	37.877	16	0.002	

## Discussion

This is the first study to assess the causality of cigarette smoking on liver fibrosis and cirrhosis using the MR analysis and GWAS. In this study, five MR analysis methods were implemented. The results indicated that a smoking history increases the risk of liver fibrosis and cirrhosis, while a lack of a smoking history reduces this risk.

Liver fibrosis is a common liver disease that results in cirrhosis with the progression of fibrosis (40). The activation of hepatic stellate cells (HSCs) is a key etiological factor in liver fibrosis (41–43). Currently, liver fibrosis is assumed to be the result of pathological changes caused by an imbalance between extracellular matrix synthesis and degradation. In addition, liver tissues injured by viruses, alcohol, and other hazardous factors activate HSCs that secrete excessive extracellular matrix (ECM). The accumulation of ECM destroys the physiological architecture of the liver and leads to regression of fibrosis (44, 45).

Cigarette smoking is established as a harmful determinant of health that endangers almost all organ systems. However, the impact of cigarette smoking on the liver has been poorly studied. In recent years, the effect of cigarette smoking on the liver has attracted increasing attention (46). Long-term exposure to cigarette smoke increases the secretion of proinflammatory cytokines involved in liver cell injury (47). In addition, cigarette smoking is closely associated with non-alcoholic liver disease (NAFLD) (48). The cross-sectional study has found that increasing the daily cigarette quantity correlates with an increased incidence of fatty liver (49). A recent MR analysis identified that cigarette smoking is causally implicated in NAFLD (50). Cigarette smoking significantly increases the risk of liver fibrosis in NAFLD patients (51). Furthermore, second-hand smoking induces liver inflammation through the deregulation of genes and molecular pathways that regulate lipid metabolism (52).

This study aimed to establish a correlation between cigarette smoking and liver fibrosis and cirrhosis. To assess the causal effects, we selected six exposures, including “ever smoked,” “pack years of smoking,” “age of smoking initiation,” “smoking status: never,” “smoking status: current,” and “smoking status: previous.” Through strict statistical analysis, a positive correlation was identified between cigarette smoking and liver fibrosis and cirrhosis. “Ever smoked” and “smoking status: previous” were risk factors, whereas “smoking status: never” was seemingly protective. These results provide evidence supporting the adverse effects of cigarette smoking on liver fibrosis and cirrhosis. Cigarette smoke contains reactive oxygen species (ROS). Substantial evidence has demonstrated that ROS causes systemic oxidative damage to membrane lipids, proteins, and DNA in the human body. An imbalance between ROS and endogenous antioxidant defenses leads to oxidative stress (53–55). Several studies have shown that oxidative stress plays an important role in the development of liver fibrosis and cirrhosis (56–58). Cigarette smoking causes gut microbiota dysbiosis, which is closely associated with various diseases, including liver fibrosis (48). In this MR study, “ever smoked,” “smoking status: previous,” and “smoking status: never” rather than “smoking status: current,” “pack years of smoking,” or “age of smoking initiation” were found to be associated with liver fibrosis and cirrhosis. We speculate that it is related to the mechanisms mentioned above. This may be understood as the long-term effect of smoking on liver fibrosis and cirrhosis once it begins, and it will not stop due to changes in smoking status.

“Smoking status: previous” had positive associations with liver fibrosis and cirrhosis. The participants in the “Smoking status: previous” group had successfully quit smoking. The results indicated that smoking cessation could not reduce the risk of fibrosis and cirrhosis caused by smoking. This is an expected result because the smoking group included previous smokers who may had weight gain after smoking cessation. Both current smoking and weight gain after smoking cessation lead to a higher risk of NAFLD, which is a major cause of liver fibrosis and cirrhosis (59, 60).

The greatest advantage of this study is the use of a two-sample MR as a statistical method. Using a two-sample MR eliminates the bias caused by confounding and reverse causality issues (32). In addition, the population in this study was restricted to Europe; therefore, the bias resulting from population stratification was reduced. The exposures and outcomes were derived from different GWAS consortiums, and there was no sample overlap. Furthermore, the sample size in this study was sufficiently large to ensure the reliability of the results. Finally, five MR methods were used, and several sensitivity analyses were conducted to ensure the stability of the results.

However, limitations were present in this study. First, only the European population was assessed; therefore, the findings might not apply to other races. Second, the exposures focused on cigarette smoking habits and status, but the cigarette smoking propensity of patients was unavailable. Third, the causal effects of cigarette smoking on liver fibrosis and cirrhosis were assessed in this study, but the mechanisms underlying these effects are unknown. Fourth, cigarette filter types were not classified. Nowadays, many people have the habit of using electronic cigarettes, but this study could not observe the effects of electronic cigarettes on liver fibrosis and cirrhosis. Finally, numerous unhealthy lifestyle habits, such as drinking alcohol, lack of exercise, and unhealthy diet, are related to liver fibrosis and cirrhosis. However, the participants in this program of the Integrative Epidemiology Unit GWAS database were not grouped based on whether they had these bad habits or not, which led to our study only being able to observe the effects of smoking on liver fibrosis and cirrhosis and being unable to explore the mutual effects of these bad habits and smoking on liver fibrosis and cirrhosis. Future studies should strive to address these gaps.

## Conclusion

This MR study provides evidence supporting the causal effects of cigarette smoking on liver fibrosis and cirrhosis. Cigarette smoking is a harmful determinant of health, and strict avoidance of cigarette smoking reduces the incidence of liver fibrosis and cirrhosis.

## Data availability statement

The datasets presented in this study can be found in online repositories. The names of the repository/repositories and accession number(s) can be found in the article/Supplementary material.

## Author contributions

LG: Writing – original draft, Formal analysis, Investigation, Validation. YA: Validation, Writing – review & editing. XH: Data curation, Formal analysis, Writing – review & editing. WL: Data curation, Formal analysis, Writing – review & editing. FC: Investigation, Writing – review & editing. YF: Writing – review & editing. SG: Writing – review & editing. LH: Writing – review & editing. KW: Investigation, Resources, Supervision, Validation, Writing – review & editing.

## References

[1] Asrani SK Devarbhavi H Eaton J Kamath PS. Burden of liver diseases in the world. J Hepatol. (2019) 70:151–71. doi: 10.1016/j.jhep.2018.09.014 30266282

[2] Llovet JM Zucman-Rossi J Pikarsky E Sangro B Schwartz M Sherman M. Hepatocellular Carcinoma. Nat Rev Dis Primers. (2016) 2:16018. doi: 10.1038/nrdp.2016.18 27158749

[3] Roehlen N Crouchet E Baumert TF. Liver fibrosis: mechanistic concepts and therapeutic perspectives. Cells. (2020) 9:875. doi: 10.3390/cells9040875, PMID: 32260126 PMC7226751

[4] Thomson NC Polosa R Sin DD. Cigarette smoking and asthma. J Allergy Clin Immunol Pract. (2022) 10:2783–97. doi: 10.1016/j.jaip.2022.04.034 35533997

[5] Kim NH Jung YS Hong HP Park JH Kim HJ Park DI. Association between cotinine-verified smoking status and risk of nonalcoholic fatty liver disease. Liver Int. (2018) 38:1487–94. doi: 10.1111/liv.13701, PMID: 29359396

[6] Bai X Wei H Liu W Coker OO Gou H Liu C. Cigarette smoke promotes colorectal Cancer through modulation of gut microbiota and related metabolites. Gut. (2022) 71:2439–50. doi: 10.1136/gutjnl-2021-325021 35387878 PMC9664112

[7] Larsson SC Burgess S Michaëlsson K. Smoking and stroke: a Mendelian randomization study. Ann Neurol. (2019) 86:468–71. doi: 10.1002/ana.25534, PMID: 31237718 PMC6701987

[8] Bauer-Kemény C Herth F. Smoking-toxic substances and immunological consequences. Radiologie. (2022) 62:731–7. doi: 10.1007/s00117-022-01006-6 35925095

[9] Sales MPU Araújo AJ Chatkin JM Godoy I Pereira LFF Castellano MVCO. Update on the approach to smoking in patients with respiratory diseases. J Bras Pneumol. (2019) 45:e20180314. doi: 10.1590/1806-3713/e20180314, PMID: 31271604 PMC6715029

[10] Gan H Hou X Zhu Z Xue M Zhang T Huang Z. Smoking: a leading factor for the death of chronic respiratory diseases derived from global burden of disease study 2019. BMC Pulm Med. (2022) 22:1–11. doi: 10.1186/s12890-022-01944-w, PMID: 35443660 PMC9019969

[11] DiGiacomo SI Jazayeri M-A Barua RS Ambrose JA. Environmental tobacco smoke and cardiovascular disease. Int J Environ Res Public Health. (2019) 16:96. doi: 10.3390/ijerph16010096, PMID: 30602668 PMC6339042

[12] Kondo T Nakano Y Adachi S Murohara T. Effects of tobacco smoking on cardiovascular disease. Circ J. (2019) 83:1980–5. doi: 10.1253/circj.CJ-19-0323 31462607

[13] Berkowitz L Schultz BM Salazar GA Pardo-Roa C Sebastián VP Álvarez-Lobos MM. Impact of cigarette smoking on the gastrointestinal tract inflammation: opposing effects in Crohn’s disease and ulcerative colitis. Front Immunol. (2018) 9:74. doi: 10.3389/fimmu.2018.00074, PMID: 29441064 PMC5797634

[14] Hecht SS Hatsukami DK. Smokeless tobacco and cigarette smoking: chemical mechanisms and Cancer prevention. Nat Rev Cancer. (2022) 22:143–55. doi: 10.1038/s41568-021-00423-4, PMID: 34980891 PMC9308447

[15] Pang Q Qu K Zhang J Xu X Liu S Song S. Cigarette smoking increases the risk of mortality from liver Cancer: a clinical-based cohort and Meta-analysis. J Gastroenterol Hepatol. (2015) 30:1450–60. doi: 10.1111/jgh.12990, PMID: 25967392

[16] Lee Y-CA Cohet C Yang Y-C Stayner L Hashibe M Straif K. Meta-analysis of epidemiologic studies on cigarette smoking and liver Cancer. Int J Epidemiol. (2009) 38:1497–511. doi: 10.1093/ije/dyp280, PMID: 19720726

[17] Bowden J Holmes MV. Meta-analysis and Mendelian randomization: a review. Res Synth Methods. (2019) 10:486–96. doi: 10.1002/jrsm.1346, PMID: 30861319 PMC6973275

[18] Davey Smith G Hemani G. Mendelian randomization: genetic anchors for causal inference in epidemiological studies. Hum Mol Genet. (2014) 23:R89–98. doi: 10.1093/hmg/ddu328, PMID: 25064373 PMC4170722

[19] Davies NM Holmes MV Smith GD. Reading Mendelian randomisation studies: a guide, glossary, and checklist for clinicians. BMJ. (2018) 362:k601. doi: 10.1136/bmj.k601, PMID: 30002074 PMC6041728

[20] Emdin CA Khera AV Kathiresan S. Mendelian randomization. JAMA. (2017) 318:1925–6. doi: 10.1001/jama.2017.17219 29164242

[21] Jones DS Podolsky SH. The history and fate of the gold standard. Lancet. (2015) 385:1502–3. doi: 10.1016/S0140-6736(15)60742-5 25933270

[22] Bothwell LE Podolsky SH. The emergence of the randomized, controlled trial. N Engl J Med. (2016) 375:501–4. doi: 10.1056/NEJMp1604635 27509097

[23] Bennett DA Holmes MV. Mendelian randomisation in cardiovascular research: an introduction for clinicians. Heart. (2017) 103:1400–7. doi: 10.1136/heartjnl-2016-310605, PMID: 28596306 PMC5574403

[24] Gupta V Walia G Sachdeva M. ‘Mendelian randomization’: an approach for exploring causal relations in epidemiology. Public Health. (2017) 145:113–9. doi: 10.1016/j.puhe.2016.12.033 28359378

[25] Gala H Tomlinson I. The use of Mendelian randomisation to identify causal Cancer risk factors: promise and limitations. J Pathol. (2020) 250:541–54. doi: 10.1002/path.5421, PMID: 32154591

[26] Tin A Köttgen A. Mendelian Randomization Analysis as a Tool to Gain Insights into Causes of Diseases: A Primer. J Am Soc Nephrol. (2021) 32:2400–7. doi: 10.1681/ASN.2020121760, PMID: 34135084 PMC8722812

[27] Richmond RC. Davey Smith G. Mendelian Randomization: Concepts and Scope. Cold Spring Harb Perspect Med. (2022) 12:a040501. doi: 10.1101/cshperspect.a040501, PMID: 34426474 PMC8725623

[28] Ference BA Holmes MV Smith GD. Using Mendelian Randomization to Improve the Design of Randomized Trials. Cold Spring Harb Perspect Med. (2021) 11:a040980. doi: 10.1101/cshperspect.a040980, PMID: 33431510 PMC8247560

[29] Zhou W Liu G Hung RJ Haycock PC Aldrich MC Andrew AS. Causal relationships between body mass index, smoking and lung Cancer: Univariable and multivariable Mendelian randomization. Int J Cancer. (2021) 148:1077–86. doi: 10.1002/ijc.33292, PMID: 32914876 PMC7845289

[30] Tang H Yang D Han C Mu P. Smoking, DNA methylation, and breast Cancer: a Mendelian randomization study. Front Oncol. (2021) 11:745918. doi: 10.3389/fonc.2021.745918, PMID: 34650928 PMC8507148

[31] Xiong J Yang L Deng YQ Yan SY Gu JM Li BH. The causal association between smoking, alcohol consumption and risk of bladder Cancer: a Univariable and multivariable Mendelian randomization study. Int J Cancer. (2022) 151:2136–43. doi: 10.1002/ijc.34228, PMID: 35904850

[32] Richmond RC Davey SG. Commentary: orienting causal relationships between two phenotypes using bidirectional Mendelian randomization. Int J Epidemiol. (2019) 48:907–11. doi: 10.1093/ije/dyz149, PMID: 31298278

[33] Bowden J Davey Smith G Burgess S. Mendelian randomization with invalid instruments: effect estimation and Bias detection through egger regression. Int J Epidemiol. (2015) 44:512–25. doi: 10.1093/ije/dyv080, PMID: 26050253 PMC4469799

[34] Bowden J Del Greco MF Minelli C Davey Smith G Sheehan NA Thompson JR. Assessing the suitability of summary data for two-sample Mendelian randomization analyses using Mr-egger regression: the role of the I2 statistic. Int J Epidemiol. (2016) 45:1961–74. doi: 10.1093/ije/dyw220, PMID: 27616674 PMC5446088

[35] Burgess S Small DS Thompson SG. A review of instrumental variable estimators for Mendelian randomization. Stat Methods Med Res. (2017) 26:2333–55. doi: 10.1177/0962280215597579, PMID: 26282889 PMC5642006

[36] Duan C Shi J Yuan G Shou X Chen T Zhu X. Causal association between heart failure and Alzheimer’s disease: a two-sample bidirectional Mendelian randomization study. Front Genet. (2022) 12:772343. doi: 10.3389/fgene.2021.772343, PMID: 35087565 PMC8787319

[37] Burgess S Butterworth A Thompson SG. Mendelian randomization analysis with multiple genetic variants using summarized data. Genet Epidemiol. (2013) 37:658–65. doi: 10.1002/gepi.21758, PMID: 24114802 PMC4377079

[38] Verbanck M Chen C-Y Neale B Do R. Detection of widespread horizontal pleiotropy in causal relationships inferred from Mendelian randomization between complex traits and diseases. Nat Genet. (2018) 50:693–8. doi: 10.1038/s41588-018-0099-7 29686387 PMC6083837

[39] Burgess S Thompson SG. Interpreting findings from Mendelian randomization using the Mr-egger method. Eur J Epidemiol. (2017) 32:377–89. doi: 10.1007/s10654-017-0255-x, PMID: 28527048 PMC5506233

[40] Parola M Pinzani M. Liver fibrosis: pathophysiology, Pathogenetic targets and clinical issues. Mol Asp Med. (2019) 65:37–55. doi: 10.1016/j.mam.2018.09.002, PMID: 30213667

[41] Zhang C-Y Yuan W-G He P Lei J-H Wang C-X. Liver fibrosis and hepatic stellate cells: etiology, pathological hallmarks and therapeutic targets. World J Gastroenterol. (2016) 22:10512–22. doi: 10.3748/wjg.v22.i48.10512, PMID: 28082803 PMC5192262

[42] Elpek GÖ. Cellular and molecular mechanisms in the pathogenesis of liver fibrosis: an update. World J Gastroenterol. (2014) 20:7260–76. doi: 10.3748/wjg.v20.i23.7260, PMID: 24966597 PMC4064072

[43] Nianan L Jiangbin L Yu W Jianguo L Rui D. Hepatic stellate cell: a double-edged sword in the liver. Physiol Res. (2021) 70:821–9. doi: 10.33549/physiolres.934755, PMID: 34717063 PMC8815467

[44] Caligiuri A Gentilini A Pastore M Gitto S Marra F. Cellular and molecular mechanisms underlying liver fibrosis regression. Cells. (2021) 10:2759. doi: 10.3390/cells10102759, PMID: 34685739 PMC8534788

[45] Dawood RM El-Meguid MA Salum GM El Awady MK. Key players of hepatic fibrosis. J Interf Cytokine Res. (2020) 40:472–89. doi: 10.1089/jir.2020.0059 32845785

[46] Marti-Aguado D Clemente-Sanchez A Bataller R. Cigarette smoking and liver diseases. J Hepatol. (2022) 77:191–205. doi: 10.1016/j.jhep.2022.01.016 35131406

[47] Moszczyński P Żabiński Z Moszczyński P Jr Rutowski J Słowiński S Tabarowski Z. Immunological findings in cigarette smokers. Toxicol Lett. (2001) 118:121–7. doi: 10.1016/s0378-4274(00)00270-8 11137318

[48] Chen B Sun L Zeng G Shen Z Wang K Yin L. Gut Bacteria alleviate smoking-related Nash by degrading gut nicotine. Nature. (2022) 610:562–8. doi: 10.1038/s41586-022-05299-4, PMID: 36261549 PMC9589931

[49] Jung H-S Chang Y Kwon M-J Sung E Yun KE Cho YK. Smoking and the risk of non-alcoholic fatty liver disease: a cohort study. Am J Gastroenterol. (2019) 114:453–63. doi: 10.1038/s41395-018-0283-5 30353055

[50] Yuan S Chen J Li X Fan R Arsenault B Gill D. Lifestyle and metabolic factors for nonalcoholic fatty liver disease: Mendelian randomization study. Eur J Epidemiol. (2022) 37:723–33. doi: 10.1007/s10654-022-00868-3, PMID: 35488966 PMC9329390

[51] Ou H Fu Y Liao W Zheng C Wu X. Association between smoking and liver fibrosis among patients with nonalcoholic fatty liver disease. Can J Gastroenterol Hepatol. (2019) 2019:6028952–5. doi: 10.1155/2019/6028952, PMID: 31737583 PMC6815556

[52] Tommasi S Yoon J-I Besaratinia A. Secondhand smoke induces liver steatosis through deregulation of genes involved in hepatic lipid metabolism. Int J Mol Sci. (2020) 21:1296. doi: 10.3390/ijms21041296, PMID: 32075112 PMC7072934

[53] Caliri AW Tommasi S Besaratinia A. Relationships among smoking, oxidative stress, inflammation, macromolecular damage, and Cancer. Mutat Res Rev Mutat Res. (2021) 787:108365. doi: 10.1016/j.mrrev.2021.108365, PMID: 34083039 PMC8287787

[54] Prasad S Gupta SC Tyagi AK. Reactive oxygen species (Ros) and Cancer: role of Antioxidative nutraceuticals. Cancer Lett. (2017) 387:95–105. doi: 10.1016/j.canlet.2016.03.042 27037062

[55] Sahoo BM Banik BK Borah P Jain A. Reactive oxygen species (Ros): key components in Cancer therapies. Anti Cancer Agents Med Chem. (2022) 22:215–22. doi: 10.2174/1871520621666210608095512 34102991

[56] Yi J Wu S Tan S Qin Y Wang X Jiang J. Berberine alleviates liver fibrosis through inducing ferrous redox to activate Ros-mediated hepatic stellate cells Ferroptosis. Cell Death Dis. (2021) 7:374. doi: 10.1038/s41420-021-00768-7, PMID: 34864819 PMC8643357

[57] Xu Y Chen J Jiang W Zhao Y Yang C Wu Y. Multiplexing Nanodrug ameliorates liver fibrosis via Ros elimination and inflammation suppression. Small. (2022) 18:e2102848. doi: 10.1002/smll.202102848, PMID: 34758098

[58] Luangmonkong T Suriguga S Mutsaers HA Groothuis GM Olinga P Boersema M. Targeting oxidative stress for the treatment of liver fibrosis. Rev Physiol Biochem Pharmacol. (2018) 175:71–102. doi: 10.1007/112_2018_10 29728869

[59] Jeong S Oh YH Choi S Chang J Kim SM Park SJ. Association of Change in smoking status and subsequent weight change with risk of nonalcoholic fatty liver disease. Gut Liver. (2023) 17:150–8. doi: 10.5009/gnl220038, PMID: 36325764 PMC9840925

[60] Han S Jeong S Ahn JC Cho Y Choi S Park SJ. Association of post-smoking cessation changes in fasting serum glucose with changes in predicted fatty liver score. Sci Rep. (2023) 13:10300. doi: 10.1038/s41598-023-37194-x 37365204 PMC10293240

