# Supplementary figures and images for: A Mendelian randomization study on the causal effects of cigarette smoking on liver fibrosis and cirrhosis
Source: Front Med (Lausanne). 2024 May 22;11:1390049. doi: 10.3389/fmed.2024.1390049 (PMC11150551; doi:10.3389/fmed.2024.1390049)

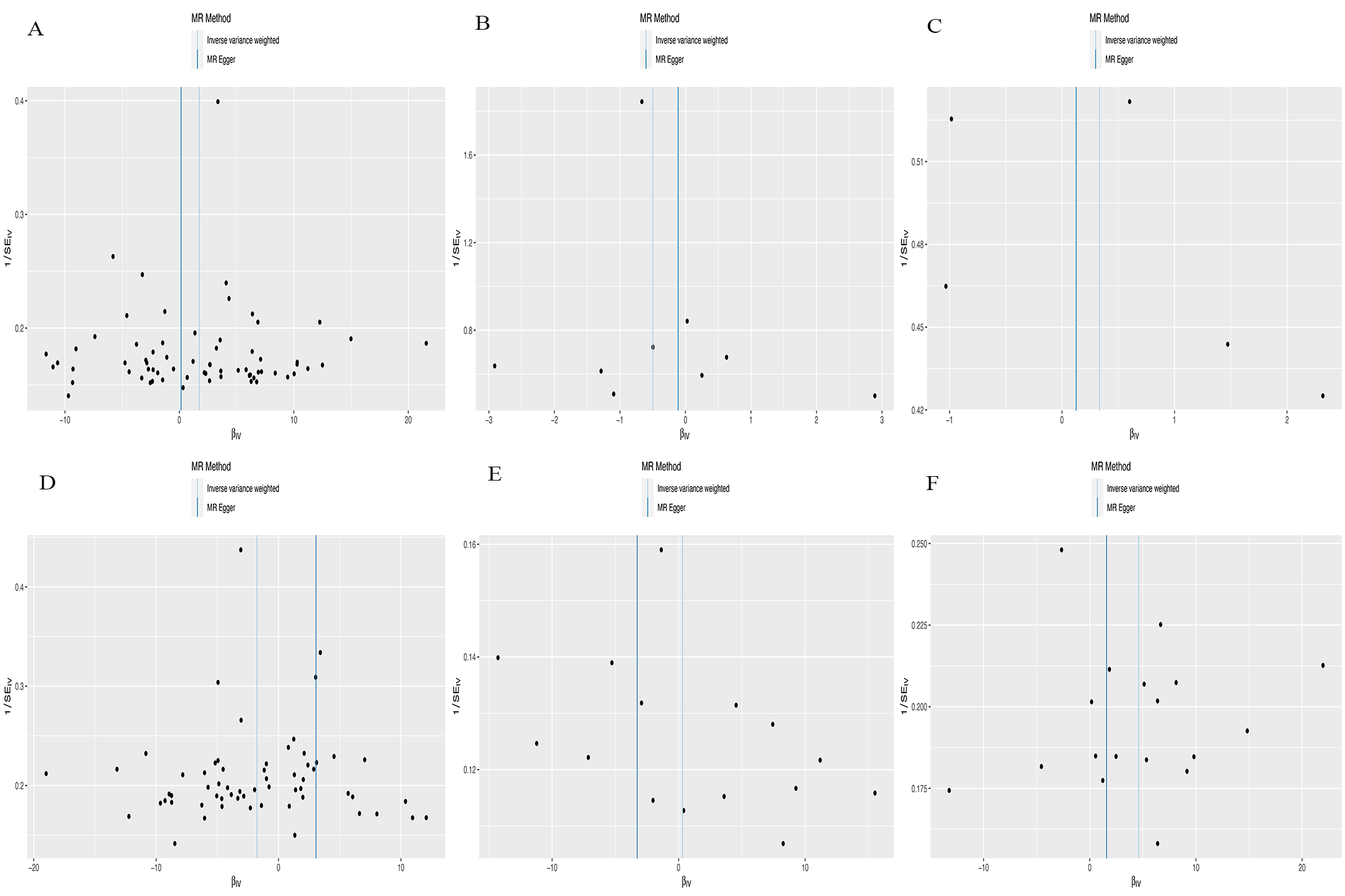

Supplement: Supplementary file 1 [file Data_Sheet_1.ZIP › Supplementary Files/Supplementary Figure 1 .tif]
